# The superposition effects of air pollution on government health expenditure in China— spatial evidence from GeoDetector

**DOI:** 10.1186/s12889-022-13702-y

**Published:** 2022-07-25

**Authors:** Qi Xia, Xiyu Zhang, Yanmin Hu, Wanxin Tian, Wenqing Miao, Bing Wu, Yongqiang Lai, Jia Meng, Zhixin Fan, Chenxi Zhang, Ling Xin, Jingying Miao, Qunhong Wu, Mingli Jiao, Linghan Shan, Nianshi Wang, Baoguo Shi, Ye Li

**Affiliations:** 1 Health Policy and Hospital Management Research Center, School of Health Management, Harbin Medical University, Harbin, 150086 Heilongjiang China; 2 School of Public Health, Harbin Medical University, Harbin, 150086 Heilongjiang China; 3 Heilongjiang Provincial Hospital, Harbin, 150086 Heilongjiang China; 4 The 2nd Affiliated Hospital of Harbin Medical University, Harbin, 150086, Heilongjiang China; 5 Department of Social Medicine, Harbin Medical University, Harbin, 150086 Heilongjiang China; 6 The Department of Hospital Offices, the affiliated Wuxi No.2 People’s Hospital of Nanjing Medical University, Liangxi District, Wuxi, 214002 Jiangsu China; 7 Department of Economics, School of Economics, Minzu University of China, No.27 Zhongguancun South Avenue, Beijing, 100081 China

**Keywords:** Air pollution, Government health expenditure, GeoDetector

## Abstract

**Background:**

As the fifth-largest global mortality risk factor, air pollution has caused nearly one-tenth of the world’s deaths, with a death toll of 5 million. 21% of China’s disease burden was related to environmental pollution, which is 8% higher than the US. Air pollution will increase the demand and utilisation of Chinese residents’ health services, thereby placing a greater economic burden on the government. This study reveals the spatial impact of socioeconomic, health, policy and population factors combined with environmental factors on government health expenditure.

**Methods:**

Spearman’s correlation coefficient and GeoDetector were used to identify the determinants of government health expenditure. The GeoDetector consist of four detectors: factor detection, interaction detection, risk detection, and ecological detection. One hundred sixty-nine prefecture-level cities in China are studied. The data sources are the 2017 data from China’s Economic and Social Big Data Research Platform and WorldPOP gridded population datasets.

**Results:**

It is found that industrial sulfur dioxide attributed to government health expenditure, whose q value (explanatory power of X to Y) is 0.5283. The interaction between air pollution factors and other factors will increase the impact on government health expenditure, the interaction value (explanatory power of × 1∩× 2 to Y) of GDP and industrial sulfur dioxide the largest, whose values is 0.9593. There are 96 simple high-risk areas in these 169 areas, but there are still high-risk areas affected by multiple factors.

**Conclusion:**

First, multiple factors influence the spatial heterogeneity of government health expenditure. Second, health and socio-economic factors are still the dominant factors leading to increased government health expenditure. Third, air pollution does have an important impact on government health expenditure. As a catalytic factor, combining with other factors, it will strengthen their impact on government health expenditure. Finally, an integrated approach should be adopted to synergisticly governance the high-risk areas with multi-risk factors.

## Background

As the fifth-largest global mortality risk factor, air pollution has caused nearly one-tenth of the world’s deaths, with a death toll of 5 million [1]. Some scholars have shown that every 10,000 tons of industrial sulfur dioxide emissions in cities will lead to an increase in lung cancer and respiratory disease deaths by 0.035 and 0.030 per 10,000 people, respectively [2]. The total number of premature deaths due to PM_2.5_-exposure across China in 2013 reached 1.37 million [3] and predicted that the number of deaths could reach 2.3 million by 2030 [4]. The World Health Organization has preliminarily estimated that 21% of China’s disease burden was related to environmental pollution, which is 8% higher than that of the United States. Moreover, for every 1% increase in PM_2.5_, household health care expenditure will increase by 2.942% [5]. This would exacerbate an already-problematic situation, given that the total medical expenses of clinic visits for respiratory diseases in China had already reached an estimated 17.2–57 billion Yuan in 2014 [6]. As such, it is not difficult to see that air pollution will increase the demand and utilisation of Chinese residents’ health services, thereby placing a greater economic burden on the government.

Existing studies have proved that environmental, socioeconomic, health and other factors are affecting government health expenditure to varying degrees. First, industrial sulfur dioxide has been considered a representative air pollutant by the Asian Development Bank in terms of environmental factors. The impact of sulfur dioxide (SO_2_) on human beings has been fully proved – long-term inhalation of SO_2_ can cause chronic bronchitis, chronic rhinitis, and other diseases [7]. Moreover, adverse weather factors have increased the risk of disease – for example, the population is at a higher risk of disease in the year of drought, leading to increased health expenditure by between 9 and 17% of total consumption [8]. Extreme high temperatures will increase the number of inpatients and deaths, further affecting the government’s health expenditure [9]. Second, socioeconomic factors will also have an impact upon health expenditure – for example, certain studies have shown that with every 1% increase in per capita gross domestic product (GDP), health expenditure will increase by 0.332% [10]. Furthermore, a 1% increase in the level of urbanisation will lead to a 0.378% increase in government health expenditure within the affected region [11]. In addition, from 2008 to 2017, the ageing problem was increasingly serious; at this stage, the share of government health expenditure increased from 5.7 to 7.5% [12, 13]. Third, health factors have a natural driving effect on government health expenditure. With every 1% increase in the number of beds, the health expenditure will increase by 0.264% [11]. In sum, social, health, policy, and environmental factors all impact government health expenditure to varying degrees.

However, most of the existing literature is limited to the impact of a single dimension on government health expenditure [14, 15]. Few studies have examined the influence of air pollution on the government health expenditure – particularly from a multi-dimensional perspective via the superimposition of air pollution with social, health, environmental, and policy factors. Moreover, research regarding the spatial differentiation between air pollution and government health expenditure is still relatively nascent. And only few articles focused on the spatial difference of health expenditure; in China as caused by air pollution, albeit at the provincial level [11].

Based on the above hypothesis, this study verified spatial heterogeneity of various factors and their coupling on government health expenditure from the perspective of multi-dimensional factors. As such, we have tried to address the gap in the body of research regarding these topics. Thus, our study contributed to the existing literature in two aspects. First, we introduced a new method – the GeoDetector – to analyse the spatial heterogeneity of government health expenditure, and its driving factors, in Chinese prefecture-level cities. The method’s advantage is that it allows for identifying spatial similarities between dependent variables and independent variables and even allows for detecting an interaction between driving factors. The method’s q-value statistics is used to describe the extent to which independent variables can account for dependent variables and, thus, carries an exact physical meaning with no linear hypothesis. Second, environmental factors were introduced into the model in our study. Little attention has been paid to the impact of socioeconomic, health, environmental and policy factors on governments’ health expenditure in China at prefecture-city level. Considering a number of possible known factors, our study quantified the impact on health expenditure of prefecture-level cities in China.

## Methods

### Data source and variable screening

Based on previous studies, we constructed a model of the impact of air pollution on government health expenditure, using the latter as the dependent variable. Government health expenditure concerns governments’ funds at all levels for health services, medical security subsidies, health security administration and other health-related undertakings. Therefore, using government health expenditure as a dependent variable can lead to a more comprehensive evaluation of the government’s investment in health. This study focused on the impact of air pollution on government health expenditure, and whether the impact of socioeconomic, health and policy factors on government health expenditure has changed under the superposition of air pollution factors.

According to the “China Statistical Yearbook – 2018”, there are 294 prefecture-level cities, and 4 municipalities, directly under the purview of the central government. Due to a lack of data availability for many of these cities, the data for 200 prefecture-level cities and 4 municipalities were collected finally. The indexed data were mainly collected via China’s Economic and Social Big Data Research Platform, including GDP, urbanisation level (UL), proportion of secondary industry (PSI), the number of hospital beds (NHB), the number of hospitals (NH), the number of (assistant) doctors (ND), integrating medical insurance reform (IURMI), the proportion of government health expenditure in GDP (PGH), annual average temperature (AT), annual rainfall (AR), industrial sulfur dioxide emissions (ISDE) and population density (PD) **(**Table 1**)**. It should be noted that PD was taken from WorldPOP gridded population datasets and further corrected according to yearbook demographic data. This population remote-sensing dataset has been widely used to estimate the spatial distribution of the population, as can be found in much of the literature [16].

**Table 1:** Descriptions of the indicators for influencing factors

Respects	Variable	Code	Unit	Data sources
Dependent variable	Government health expenditure	GHE	10^4^ Yuan	the Statistical Yearbook of the prefecture-level cities in 2017
Socioeconomic factors	Gross Domestic Product	GDP	10^8^ Yuan	the Statistical Yearbook of the prefecture-level cities in 2017
	Urbanisation level	UL	Percent	the Statistical Yearbook of the prefecture-level cities in 2017
	Proportion of Secondary Industry	PSI	Percent	China Urban Statistical Yearbook – 2018
Health factors	Number of Hospital Beds	NHB	Beds	China Urban Statistical Yearbook – 2018
	Number of hospitals	NH	Hospitals	the Statistical Yearbook of the prefecture-level cities in 2017
	Number of doctors	ND	Person	China Urban Statistical Yearbook – 2018
Policy factors	Integration of urban and rural residents’ medical insurance	IURMI	/	Human resources and social security websites of cities
	Proportion of government health care expenditure in GDP	PGH	Percent	the Statistical Yearbook of the prefecture-level cities in 2017
Environmental factors	Annual average temperature	AT	Centigrade	the Statistical Yearbook of the prefecture-level cities in 2017
	Annual rainfall	AR	Millimeter	the Statistical Yearbook of the prefecture-level cities in 2017
	Industrial sulfur dioxide Emissions	ISDE	10^4^ Tons	China Urban Statistical Yearbook – 2018
Population factor	Population density	PD	10^4^ person per square kilometer	WorldPOP gridded population datasets

### Spearman’s correlation coefficient

Spearman’s correlation coefficient is used to measure the dependency of two variables by quantifying the relationship between government health expenditure and related influencing factors, thereby determining whether the relationship is positive or negative. The method uses a monotone equation to evaluate the correlation between two statistical variables. In this study, we used a bivariate association analysis of bilateral tests. The formula for the correlation coefficient, ρ, is as follows:

1 $$\rho =\frac{\sum_i\left({x}_i-\overline{x}\right)\left({y}_i-\overline{y}\right)}{\sqrt{\sum_i{\left({x}_i-\overline{x}\right)}^2{\sum}_i{\left({y}_i-\overline{y}\right)}^2}}$$

In this instance, the value ρ represents the association between government health expenditure and each influencing factor – with a range of [− 1, 1]. A positive value indicates a positive correlation between two variables, whereas a negative value indicates a negative correlation. Furthermore, larger values indicate stronger correlations. The dependent variable, Y, represents government health expenditure, while the independent variable, X, represents the influencing factor of the GeoDetector. We used this method to evaluate the dependence of government health expenditure on influencing factors. The tool used to calculate the Spearman correlation coefficient was IBM’s SPSS statistics package (version 19).

### The GeoDetector method (GDM)

In this study, the impact of 11 driving factors on Chinese government health expenditure was measured via the GeoDetector. GeoDetector is a spatial statistical method for detecting spatial heterogeneity, quantifying driving factors and their interactions. Its basic principle concerns the division of the study area into several sub-regions. If the intra-layer variance is less than the inter-layer variance, there will be spatial heterogeneity. Compared with the traditional linear models, GeoDetector can detect both qualitative and quantitative data without considering the assumptions of either linearity or collinearity. However, the detection of continuous data needs to be translated into discrete qualitative data – the difficulty lies in the discretisation of continuous data via the appropriate methods, which determines the discretisation method and interval range of continuous data at different levels. Then, factor detection and interaction detection were used to calculate the q value and interaction q value respectively after continuous discretisation data. By comparing the q value and interactive q value of different levels of discretisation methods, the optimal discretisation method is finally determined [17].

In this study, Jenks Natural Breaks Classification method was used to classify the continuous data into discrete categories. According to the interval value, the 10 numerical influencing factors were classified along 7 natural breakpoints, while the regions were arranged in ascending order. The “1” sub-region is the minimum interval value, whereas the “7” sub-region is the maximum interval value. In addition, Sun adopted the standard of 10 * 10 km [18]. Further since GeoDetector software can accommodate 32,767 at most [19], we finally adopted 20 * 20 km areas. ArcGIS 10.2 was used to delimit the administrative regions of China in 20 km*20 km areas. Subsequently, information regarding the independent and dependent variables of each grid point’s location was removed to make the variable information of the grid point. These variables were input into GeoDetector.

GeoDetector consists of four detectors: factor detection, interaction detection, risk detection, and ecological detection [19].

1. Factor detection is used to detect the degree of explanation of driving factors for spatial differentiation of government health expenditure. The use of q allows for the value to be measured, whose expression is:

2 $$\mathrm{q}=1-\frac{\sum_{\mathrm{h}=1}^{\mathrm{L}}{N}_h{\sigma}_h^2}{N{\sigma}^2}=1-\frac{SSW}{SST}$$

3 $$\mathrm{SSW}={\sum}_{\mathrm{h}=1}^{\mathrm{L}}{N}_h{\sigma}_h^2, SST=N{\sigma}^2$$

Where: h = 1…; L = the Strata of government health expenditure, or impact factor X; *N*_*h*_ and N are layer h and the number of units in the whole region, respectively; and *σ*^2^ are the variance of government health expenditure of layer h and the district, respectively. SSW and SST are, respectively, the sum of intra-layer variances and the total variance of the whole region. The range of q is [0, 1], which means that the influencing factor has q% explanatory power concerning government health expenditure. The larger the q value is, the stronger the impact of the influencing factor on government health expenditure will be. The value of q further represents the influencing factor x, which explains government health expenditure, y, of 100 × q %.

(2) Interaction detection evaluates whether the interactive effect of different factors × 1 and × 2 will increase or decrease the explanatory power of government health expenditure. By comparing the relationships among q(× 1∩× 2), q(× 1), and q(× 2), the interaction value means whether the interactive effect of different factors X1 and X2 will increase or decrease the explanatory power of government health expenditure. The interaction results can be divided into five categories: nonlinear weaken, single-factor nonlinear weaken, two-factor enhancement, independence, and nonlinear enhancement (See Table 2). The interaction relationship is as follows:

**Table 2:** Types of interaction between two factors on dependent variables

Description	Interaction
q(× 1∩× 2) < Min(q(x), q(× 2))	Nonlinear weakening
Min(q(× 1), q(× 2)) < q(× 1∩× 2) < Max(q(× 1), q(× 2))	Single factor nonlinear weakening
q(x1∩x2) > Max(q(× 1), q(× 2))	Two factor enhancement
q(×1∩×2) = q(× 1) + q(× 2)	Independence
q(x1∩x2) > q(× 1) + q(× 2)	Nonlinear enhancement

(3) For risk detection, according to the classification of each influencing factor, the study area is divided into multiple sub-regions to identify significant differences in average government health expenditure among the sub-regions. The formula is defined as:

4 $$t_{\overline yh=1\overline yh2}=\frac{{\overline Y}_{h=1}-{\overline Y}_{h=2}}{\left[\frac{Var\left({\overline Y}_{h=1}\right)}{\eta_{h=1}}+\frac{Var\left({\overline Y}_{h=2}\right)}{\eta_{h=2}}\right]^{1/2}}$$

Where ${\overline{Y}}_h$ represents the average value of Y in the sub-region (h); is the number of samples in the sub-region (h), and Var is variance.

(4) Ecological detection determines whether the two influencing factors have significant differences in the spatial distribution of government health expenditure and is expressed as:

5 $$F=\frac{N_{X1}\left({N}_{X2}-1\right){SSW}_{X1}}{N_{X2}\left({N}_{X1}-1\right){SSW}_{X2}}$$

6 $${SSW}_X={\sum}_{\mathrm{h}=1}^{L1}{N}_{\mathrm{h}}{\sigma}_{\mathrm{h}}^2,{SST}_{X2}={\sum}_{h=1}^{L2}{N}_{\mathrm{h}}{\sigma}_h^2$$

Where, *N*_*X*1_ and *N*_*X*2_ represent the sample numbers of two factors (× 1 and × 2), respectively. *SSW*_*X*1_ and *SSW*_*X*2_ are the sum of squares of the sub-regions as generated by the factors X1 and X2, respectively. *L*1 and *L*2 represent the number of subregions of X1 and X2, respectively. The null hypothesis is defined as *h*_0_ : *SSW*_*X*1_ = *SSW*_*X*2_. The rejected *h*_0_ at the significance level *α* indicates that it is statistically significant.

### Natural breaks classification method

**Fig. 1:**
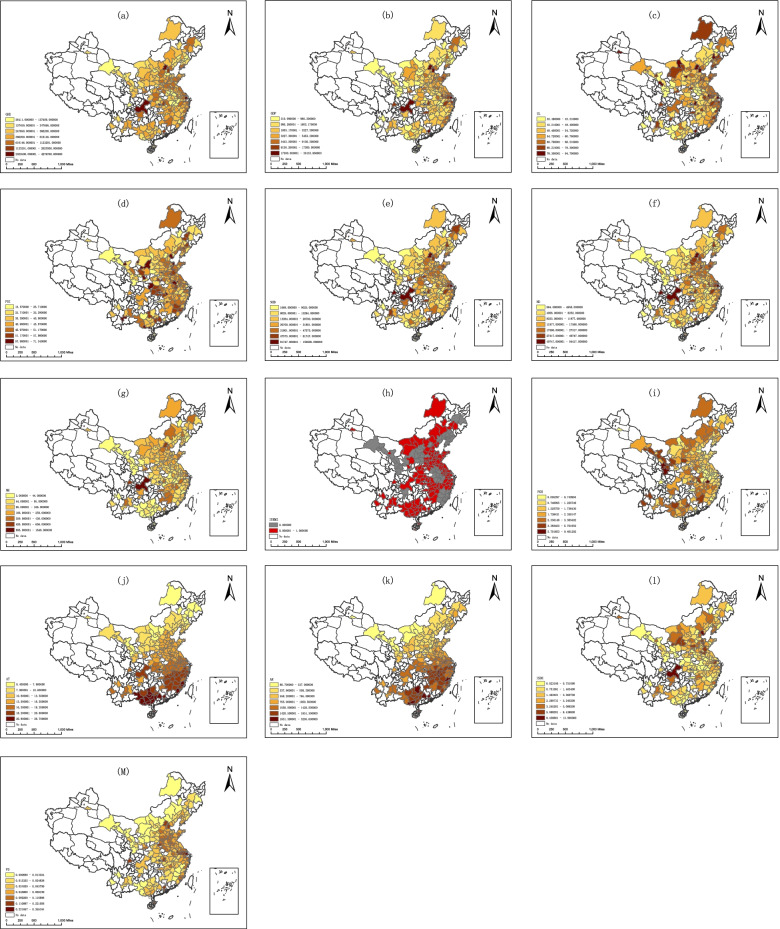
Spatial distributions of government health expenditure and influencing factors

The GeoDetector requires that continuous data be transformed into discrete data. Jenks Natural Breaks Classification was used as the classification method to optimise the layout of continuous data into “natural” categories. The basic idea of natural breaks (Jenks) is to minimise each class’s average deviation from the class’ means, and maximise each class’ deviation from the means of the other group. In other words, the method seeks to reduce the intra-class variance while maximising inter-class variance [20]. To determine the optimal classification, the Jenks Natural Breaks Classification method was used to determine the classification threshold. Because medical insurance data have been divided into two categories – “not implementing integrated medical insurance (1)” and “implementing integrated medical insurance (2)” – we used ArcGIS 10.2 software to classify the remaining 10 influencing factors used in this paper into 7 categories via the Jenks Natural Breaks Classification method. The regions were arranged in ascending order according to the interval value; the “1″ sub-area is the minimum interval value, while the “7″ sub-area is the maximum interval value – as shown in the Fig. 1.

## Results

### Spearman analysis

Spearman’s correlation coefficient was used to explore the correlation between 12 influencing factors and government health expenditure – including socioeconomic, health, policy, environmental and population factors, and to detect if the deter relationship between was positive or negative. The results showed that 6 of the 12 influencing factors were significantly and positively correlated with government health expenditure at *p* < 0.01, with influencing factors registering a *p* < 0.05. The key factors of each dimension are the number of doctors (0.784), GDP for socioeconomic factors (0.719), IURMI for policy factors (0.344), PD for population factors (0.318) and ISDE for environmental factors (0.243). The industrial sulfur dioxide emission (ISDE) of the environmental factors has a significant positive correlation (*ρ* = 0.243) with government health expenditure (See Table 3).

**Table 3:** The influencing factors and Spearman’s rho results of government health expenditure

Respects	Variable	*ρ*
Socioeconomic	GDP	0.719**
	UL	0.125
	PSI	−0.136
Health	NHB	0.775**
	NH	0.632**
	ND	0.784**
Policy	IURMI	0.344**
	PGH	0.046
Environment	AT	0.171**
	AR	0.111
	ISDE	0.243*
Population	PD	0.318^**^

** When the confidence level (double test) is 0.01, the correlation is significant

* When the confidence level (double test) is 0.05, the correlation is significant

### Factor detection analysis

The explanatory power (the q statistics) and the P value (as obtained via factor detection) are shown in Table 4. The *P* values of 11 influencing factors were all less than 0.01, indicating that the 11 influencing factors were statistically significant. The results showed that the socioeconomic, health, policy, and environmental factors of different regions impacted on government health expenditure. Among these, the top 5 key factors affecting the explanation of government health expenditure were GDP (0.8999), NHB (0.8370), ND (0.8362), NH (0.7502) and ISDE (0.5283). First, we further found that the explanatory power of GDP, NHB, and ND accounted for more than 80%, while NH exceeded 70%, indicating that the level of economic development and health resources available are key factors affecting government health expenditure. Cities with relatively developed economies and sufficient health resources contributed more government health expenditure. Second, the explanatory power of ISDE was more than 50%, indicating that it had a significant impact on government health expenditure, raising a warning which should not be ignored. The explanatory power of UL, PSI, AT, and AR was more than 10%, which indicates that AT and AR are key factors affecting government health expenditure. However, IURMI and PGH accounted for more than 5%, which, in turn, shows that IURMI and PGH will also significantly impact government health expenditure. It is noteworthy that the impact is also minimal, indicating that the health policies of prefecture-level cities in China are fair and reasonable. There is little difference across spatial units (See Table 4).

**Table 4:** The q statistics of driving factors on government health expenditure

Respects	Variable	*q*
Socioeconomic	GDP	0.8999
	UL	0.2119
	PSI	0.1034
Health	NHB	0.8370
	NH	0.7502
	ND	0.8362
Policy	IURMI	0.0277
	PGH	0.0494
Environmental	AT	0.1537
	AR	0.1350
	ISDE	0.5283
Population	PD	0.2769

### Interaction detection

The *P* values of 12 influencing factors were all less than 0.01, indicating statistical significance. Therefore, we used the interaction detection to study the explanatory power of the factors above on government health expenditure. The results showed that there are 66 pairs of interaction combinations among the 12 influencing factors – that is to say, the explanatory power of interaction between any two factors is stronger than that of any single factor. As such, some of these factors are nonlinear enhanced after interaction (expressed as #), which is the joint effect of the two factors is stronger than the sum of their independent explanatory power. For example, q (UL∩ISDE) 0.7543 > q (UL) 0.2119 + q (ISDE) 0.5283. However, more interaction combinations between some factors have a double-factor relationship (expressed as *), which indicates that the joint effect of the two factors is stronger than the maximum explanation of the two factors when independent of one another. For example, q (ISDE∩GDP) 0.9593 > q (GDP) 0.8999 > q (ISDE) 0.5283 (See Table 5).

**Table 5:** Interaction detection

	GDP	UL	PSI	NHB	ND	NH	IURMI	PGH	AT	AR	ISDE	PD
GDP	0.8999											
UL	0.9212^a^	0.2119										
PSI	0.9464^a^	0.5826^b^	0.1034									
NHB	0.9628^a^	0.9629^a^	0.8616^a^	0.8370								
ND	0.9609^a^	0.9656^a^	0.8634^a^	0.8473^a^	0.8362							
NH	0.946^a^	0.9033^a^	0.8584^b^	0.8639^a^	0.8668^a^	0.7502						
IURMI	0.9144^a^	0.2940^b^	0.1407^b^	0.8581^a^	0.8533^a^	0.8479^b^	0.0277					
PGH	0.9839^b^	0.6376^b^	0.4440^b^	0.9606^b^	0.9678^b^	0.8792^b^	0.1330^b^	0.0494				
AT	0.9282^a^	0.6373^b^	0.5627^b^	0.9154^a^	0.9138^a^	0.8996^a^	0.2245^b^	0.4657^b^	0.1537			
AR	0.9662^a^	0.6200^b^	0.4228^b^	0.9585^a^	0.9583^a^	0.8968^b^	0.1978^b^	0.5646^b^	0.3753^b^	0.1350		
ISDE	0.9593^a^	0.7543^b^	0.6296^a^	0.9022^a^	0.9075^a^	0.862^a^	0.5387^a^	0.6697^b^	0.6745^a^	0.6646^b^	0.5282	
PD	0.9184^b^	0.5916^b^	0.5508^b^	0.8747^a^	0.8713^a^	0.8552^a^	0.4606^b^	0.7951^b^	0.6318^b^	0.6112^b^	0.8815^b^	0.2769

^a^For double factor enhancement, q (X1 ∩ X2) > max (q (× 1), q (× 2))

^b^For nonlinear enhancement, q (X1 ∩ X2) > q (X1) + q (X2)

We focused on the interaction between industrial sulfur dioxide and other factors. It was found that the interaction value of GDP and ISDE is the largest, at q (GDP∩ISDE) = 0.9593.

**Fig. 2:**
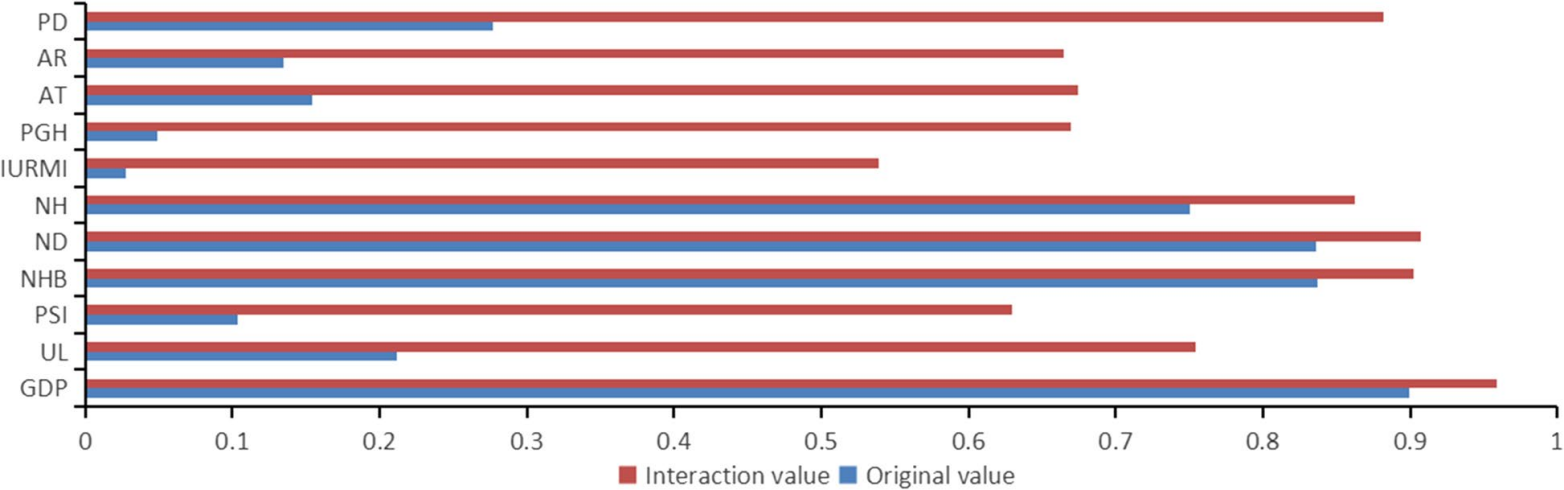
Original value q and interaction value with industrial sulfur dioxide emission

After the interaction between GDP (socioeconomic) and NHB and ND (health), and ISDE (environmental), their explanatory powers exceeded 90%, which showed a double factor enhanced relationship. We also found that, after interaction with ISDE, the q statistics of some influencing factors increased by more than 50% when compared with its own q statistics –including UL and PSI (socioeconomic), IURMI, and PGH (policy), and AT and AR (environmental) and PD (population). ISDE has a significant impact on the improvement of explanatory powers when interacted with other factors. In addition, it is noteworthy that population factors have greatly enhanced the driving force of all three health factors (double factor enhancement), as shown in Fig. 2.

### Risk detection

Through the analysis of risk detection, the average values of government health expenditure across all the sub-regions in terms of these 12 influencing factors were obtained, and the differences among the sub-regions of the influencing factors were pointed out. According to the Jenks Natural Breaks Classification method, the 12 influencing factors were divided into 7 sub-regions (in ascending order); the average value of government health expenditure, which corresponds to each sub-region, was calculated. For example, the average value of government health expenditure across the seven sub-regions in industrial sulfur dioxide was 354,972.2, 418,945, 384,154.3, 489,720.8, 439,745.7, 553,130.4, and 3,140,670. The results of the other factors were obtained using the same method.

**Fig. 3:**
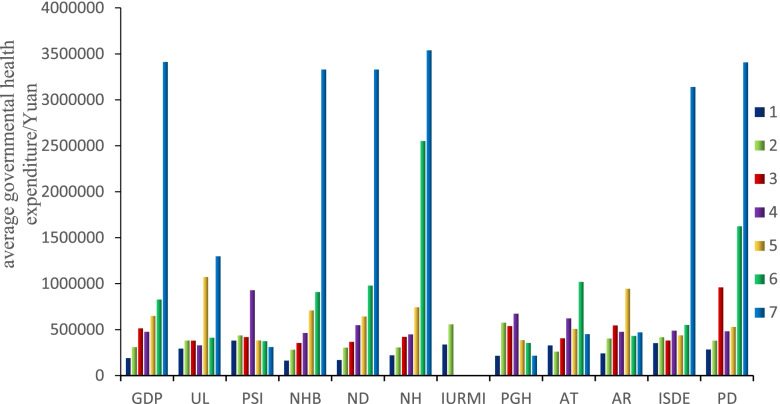
The sub-regional government health situation across each factor

As shown in the statistical chart, the average government health expenditure for each sub-region are on the rise across GDP (socioeconomic), and NHB, NH, ND, IURMI, and ISDE (environmental), as each factor increases. By comparing the government health expenditure for each factor within a sub-region, the sub-region with the highest government health expenditure was regarded as the highest-risk area. It was found that most of the high-risk areas of influencing factors are located in the seventh sub-region (See Fig. 3).

We sorted the high-risk areas according to their socioeconomic, health, policy and environmental characteristics, and summed up the 12 types of high-risk areas – namely, socioeconomic high-risk areas (10), environmental high-risk areas (12), policy high-risk areas (74), socioeconomic-health high-risk areas (1), socioeconomic-environmental high-risk areas (2), socioeconomic-policy high-risk areas (22), socioeconomic-population high-risk areas (1), policy-environment high-risk areas (31), socioeconomic-health-policy high-risk areas (1), socioeconomic-policy-environment high-risk areas (13), socioeconomic-health-policy-environment high-risk areas (1) and socioeconomic-health-policy-environment-population high-risk areas (1).

**Fig. 4:**
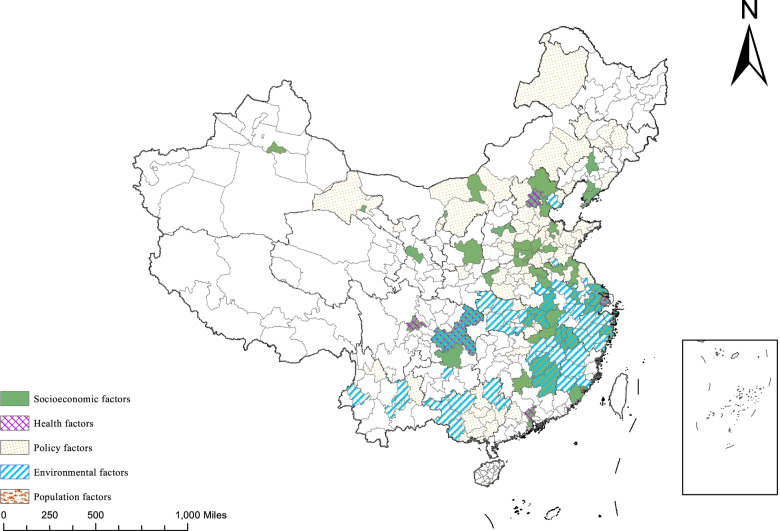
Distribution of high-risk areas

For example, Xiamen belongs to the socioeconomic-population-high-risk areas category due to the interaction between UL, PSI and PD. Beijing belongs to the socioeconomic-health high-risk area, due to the interaction of GDP and UL (socioeconomic), and NHB and ND. Tangshan is affected by the joint actions of IURMI (policy) and ISDE (environmental), categorising it as a policy-environmental high-risk area. Chongqing is a comprehensive high-risk area with a number of combined factors, such as GDP and PSI (socioeconomic); NHB, NH, ND, IURMI, and PGH (health); AT, AR, ISDE (environmental), as shown in Fig. 4.

## Discussion

Based on GeoDetector with spatial consideration, this study revealed the spatial impact of environmental factors alone, and the spatial impact of interaction between environmental factors and other ones on government health care expenditure. The following main conclusions were obtained:

### Air pollution is identified to affect government healthcare expenditure

The results of factor detection showed that industrial sulfur dioxide (environmental) accounted for 52.83% of government health expenditure, indicating that air pollution was one of the core factors affecting government health expenditure. The relationship between air pollution and government health expenditure has been previously verified by a number of scholars and is consistent with our findings [21].

**Fig. 5:**
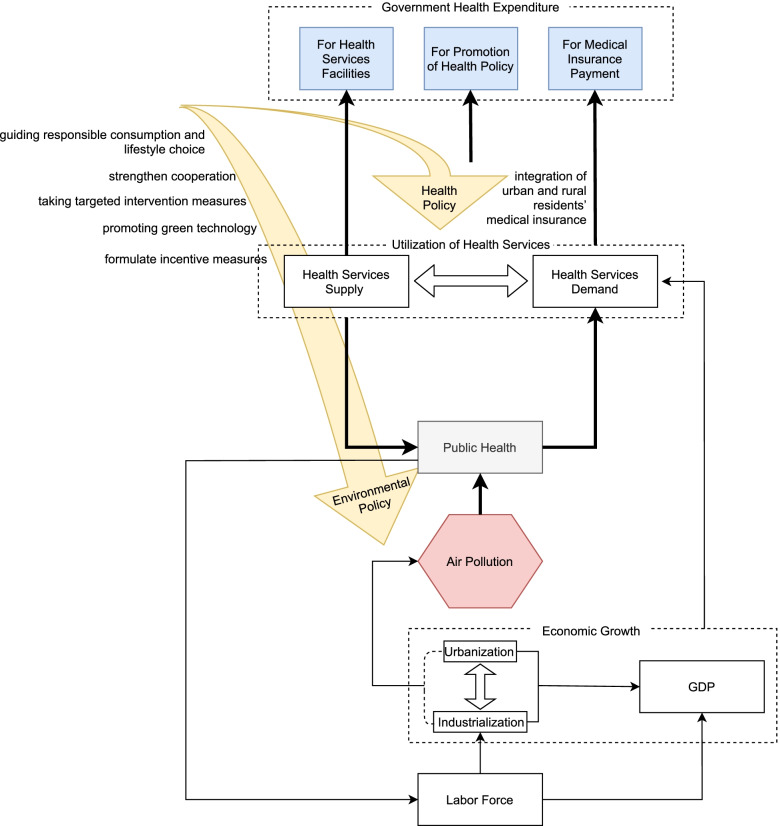
Mechanism of air pollution on government health expenditure

Figure 5 shows the impact mechanism of air pollution and various factors on government health expenditure. Air pollution has caused a wide range of threats to public health, resulting in the surge in a number of diseases – such as respiratory system, cardiovascular, and cerebrovascular diseases – thus promoting public demand for increased health services [22–27]. The demand for health services needs to be coordinated with the supply thereof – resulting in health services’ actual utilisation. Via this process, the corresponding improvement in health service allocation, the implementation of health policies, or the increase of health insurance costs (as caused by the actual utilisation of health services) will lead to government health expenditure across multiple dimensions. First, government health expenditure is increased to ensure the greater investment needed to meet residents’ growing demand and utilisation of health services – such as the investment in health service allocation and health insurance payments. In a study of urban workers in Tianjin, China, the proportion of hospitalisation expenses for respiratory diseases accounts for more than 70% of the total. In contrast, the proportion of non-individual, out of pocket payments is 68.9% [28], indicating that the health insurance system needs to bear a greater portion of the expenses. Moreover, based on the perspective of collaborative governance, more government health expenditure should be used for health policies dealing with the corresponding health problems caused by environmental pollution. As for the impact of air pollution on health expenditure, scholars have found that the spillover effect is as much as half of the total effect, suggesting that greater attention should be paid to the spatial correlation between adjacent regions [2, 29]. As Chen has indicated, if industrial sulfur dioxide emissions in a city increase by 10,000 tons, the mortality rates from lung cancer and respiratory diseases will grow by 0.217 and 1.543 per ten thousand persons, respectively, in neighbouring areas_._ Furthermore, we can also predict its impact on government health expenditure in surrounding areas. The reduction of health expenditure caused by inter-regional health reforms is eventually offset by air pollution.

Moreover, various factors will have an indirect and superimposed influence on this process at various stages. For example, there is a consensus that economic growth promotes increased demand for health services and this study tried to provide baisis for this causal chain complementation. As shown in Fig. 5, public health is an important factor in restricting the labour force, and both GDP and industrial development need the labour force as a support – that is, public health can further affect economic development through its impact on the labour force. In addition, one cannot ignore the guiding role of policy on public behaviour, potentially having a profound significance on the environment and the utilisation of health services.

The relaxation of environmental regulations can promote regional economic development over a short period, but the deteriorating ecological environment will increase the burden of government health expenditure.

As mentioned at the 68th World Health Assembly, in order to combat the health problems caused by environmental pollution, it is necessary to widely publicise healthy sector policies – such as Health in All Policies (HiAP) – and cooperate in implementing communication strategies at global, national and local levels, suggesting greater policy activity and increased levels of government health expenditure [30]. Therefore, the governance of air pollution, and its accompanying health problems, requires cooperation between regions and departments. However, although air pollution is easily spread, due to the spatial effect thereof, it is feasible and particularly important to control government expenditure.

### The superposition of air pollution factors and other factors will increase the influence on government healthcare expenditure

Although the contribution of air pollution to government healthcare expenditure is large enough, it is even more surprising that, when air pollution is combined with other factors, the contribution will experience further changes.

First, the results of the interaction detector show that the combination of certain factors is stronger than the sum of their single effects – that is to say, the combination of air pollution and another factor in the study will produce a positive synergy effect. These combinations include the level of urbanization, PGH, PD and air pollution. As shown throughout the existing literature, the level of urbanisation is proven to be one of the catalysts for air pollution [31–33]. Furthermore, the UL accounts for 16.3% of the general expenditure on health [33]. The increasing demand for health services, as brought about by urbanisation, is bound to increase the government’s healthcare investment profile. However, with the accelerated urbanisation process, the problem of environmental degradation has led to the need for additional government investment in health services as a remedy [34, 35]. If the level of carbon dioxide emissions and the degree of urbanisation increase by 1%, the need for health facilities will increase by 0.037 and 0.327%, respectively [36]– both of which would mean greater government health expenditure. In addition, the concentration of the population within cities leads to an uneven distribution of resources and an inconvenient transmission of resource information [33], contributing to difficulties experienced by residents in reaching needed health services. Furthermore, the inefficient integration of resources leads to the waste of health services, which, in essence, wastes funds allocated to expenditure on health. Unfortunately, when serious air pollution occurs, the imbalance of resource distribution between urban and rural areas is further exacerbated, with the government needing to pay more to account for the contradiction. Urbanisation is an important influencing factor in the process of air pollution’s effect on government health expenditure. Whether it is the worsening of the environment, the further expansion of air pollution, an aggravation of the inefficiency problem facing resource allocation and planning, and the intensification of the relationship between demand and supply, government health expenditure has deteriorated further, even exceeding the sum of the independent effects had by air pollution and urbanisation.

Second, the combination of air pollution with one of the factors under consideration results in a value greater than the maximum value of the two factors independently (that is, the interactive relationship is enhanced). However, the contribution of this combination is weaker than the simple addition of the two under the independent assumption. The above combinations include GDP, NHB, NH, ND, IURMI, AT, AR, and air pollution. Here we focused on the joint effect of urbanisation and air pollution on government health expenditure. GDP has been proven as being able to promote the growth of government health expenditure [15]. Moreover, GDP improves people’s living standards and greatly improves the utilisation of health services, especially in light of the negative effect of air pollution on health, thus generating an increased demand for health expenditures and a greater economic burden on the government – i.e., the growth in GDP amplifies the negative effects had by air pollution on government health expenditure. In addition, environmental factors (AT, AR) [37] have been proven to have a direct effect on health and can even affect air pollution (creating a positive feedback loop) [38]. It can be seen from the formation of acid rain that industrial sulfur dioxide emissions in the air will pose a greater threat to people’s health through rainwater [39, 40]. This could potentially explain why environmental factors make air pollution more important to public health and government health expenditure. Factors related to the supply of health services (NHB [41], NH, ND [42]) have also been confirmed to have an impact on health expenditure. There is still a gap in the current demand for health services, with the utilisation of health services unable to fully meet demand [43]. However, the emergence of air pollution increases the demand for health services and intensifies the contradiction. Therefore, more health service facilities need to be established, and more government health expenditure needs to be generated. Finally, as an integration with health policy, health insurance policies reduce the thresholds for residents to obtain health insurance protection, and help promote fair access to health services. Although air pollution increases the demand for health services of residents, the health insurance policy enables greater demands for affordable utilisation, which requires the government to increase health expenditure and share the burden of the ill-health of residents. Nevertheless, the results of this study suggest that, although these factors can increase air pollution’s weight on government expenditure, the total effect is only greater than either of them, which is not as good as UL and PGH (as discussed in the previous paragraph).

### Risk area detection and classification

According to the results of risk area detection, a total of 169 high-risk areas were found. Interestingly, there are 96 simple high-risk areas. The number of high-risk areas superimposed by mltiple factors is in the majority. These include socioeconomic-population high-risk area (1), socioeconomic-health high-risk areas (1), socioeconomic-policy high-risk areas (22), socioeconomic-environmental high-risk areas (2), policy-environmental high-risk areas (31), socioeconomic-policy-environmental high-risk areas (13), socioeconomic-health-policy high-risk areas (1), socioeconomic-health-policy-environmental high-risk areas (1), and socioeconomic-health-policy-environmental-population high-risk areas (1). To improve the cost-effectiveness ratio of government health expenditure, different measures need to be taken for different characteristics (according to the city), rather than a large number of expenses that are repeatedly incurred to make up for the adverse health outcomes caused by air pollution. First of all, for cities whose government health expenditure is only restricted by air pollution, the existing literature has proven that the air pollution in this area is a serious concern and that there is spatial spillover effect [29], which may be related to the industrial belt located throughout the region. Although industrial agglomeration areas provide employment opportunities for residents and promote the development of the local economy, it is evident that it also increases the burdens related to health expenditure for local governments. Governments should strengthen environmental infrastructure [44], implement policy control [44, 45] and pay attention to the application of clean energy in industrial production to reduce the burdens to healthcare expenditure caused by air pollution.

For high-risk areas affected by multiple factors, it is necessary to pay greater attention to the simultaneous effects of these factors’ conglomeration, not just air pollution in isolation. Several studies have confirmed the influence of social factors [46], health service factors [47], environmental factors [48], policy factors [49] and PD [50] on health services or healthcare expenditure within the context of air pollution. Taking the high-risk areas of social-health-environment-policy-population (Shanghai) as example, serious air pollution inhibits the development of the economy and, subsequently, reduces the income levels of residents. However, this general decline is more obvious among low-income groups. As a result, the gap between the rich and the poor in various air-pollution-afflicted regions is growing [51], leading to different air pollution responses and other contributing factors. Ignoring such differences will render the government’s actions meaningless. Therefore, health policy should be combined with environmental policy and urban development planning [52]. In addition, it would be helpful for further public health improvement and government health expenditure control to reduce air pollution sources, adopt intersectoral methods for setting clear health benchmarks, targets and reporting mechanisms for air pollution detection and control emerging clean energy technologies, and to treat air pollution reduction as a health-related indicator in developing sustainable development policies [30]. A large amount of government health expenditure could have greatly improved public health. However, air pollution has wasted these efforts and has, subsequently, increased the government’s burden [11], especially under the conflict between supply and demand caused by high population density. In the face of increasing health costs, a wide range of joint measures between departments, such as HiAP, can help improve the role played by government health expenditure.

## Conclusion

Using the data of China in 2017, we explored the influencing factors of air pollution on government health expenditure and its spatial governance by using GeoDetector. The results show that air pollution is indeed the explanatory factor of government health expenditure, but in this process, UL, PGH, GDP, NHB, ND, NH, IURMI, AT, AR and PD all increase this effect. In addition, in 200 prefecture-level cities and 4 municipalities, 169 regions are at high risk. Interestingly, most risk areas are driven by multiple factors. This also warns us at the policy level that measures should be taken to suit local conditions in different regions. In the areas only affected by air pollution, the government should strengthen the construction of environmental infrastructure, implement policy control, and pay attention to the application of clean energy in industrial production, so as to reduce the burden of air pollution on medical and health expenditure. But in the high-risk areas affected by multiple factors, we must pay more attention to the simultaneous influence of these factors; at the same time, a variety of joint measures, such as HiAP, should be taken among various departments to help improve the role of government health expenditure.

## Limitations and prospect

First, we use yearly cross-sectional data of 2017 to analyze the spatial heterogeneity of government health expenditure and its associated factors. Despite the use of an appropriate methodology to avoid bias as much as possible, some limitations of the cross-sectional data are difficult to resolve completely. Therefore, it is necessary to implement corresponding spatiotemporal heterogeneity studies to provide stronger supporting evidence for causality when data available. Second, this study is based only on SO2 which was proved to have strong impacts on health or health expenditure, ignoring other air pollutants. We did not use all common air pollutants in this study due to the current controversial methods for estimating the effects of air-pollutant mixtures and the poor availability of monitoring data for multiple air pollutants. This may lead to an underestimation of air pollution effects, which subsequent studies could attempt to improve when more data available and methodologies upgraded.

## Data Availability

Dataset available from the China’s Economic and Social Big Data Research Platform, https://data.cnki.net/Yearbook/Navi?type=type&code=A .
